# Common mental disorders and psychological adjustment among individuals seeking HIV testing: a study protocol to explore implications for mental health care systems

**DOI:** 10.1186/s13033-018-0196-0

**Published:** 2018-04-10

**Authors:** Jason Bantjes, Ashraf Kagee

**Affiliations:** Department of Psychology, Stellenbosch University, Matieland, Private Bag X1, Stellenbosch, 7602 South Africa

**Keywords:** HIV, Reaction to diagnosis, Common mental disorders, Mental health care systems, Protocol

## Abstract

**Background:**

In an effort to promote greater access to voluntary counseling and testing for HIV, it has become practice in many countries, including South Africa, to establish non-medical testing sites and to de-couple HIV testing from other medical and mental health care services. While it is well established that HIV infection is associated with a range of psychopathology, much of the literature has assumed that it is receipt of an HIV positive diagnosis that causes people to become depressed, traumatized, or develop other psychiatric symptoms. Empirical data about the baseline psychiatric condition and mental health care needs of persons seeking HIV testing is scarce. Understanding the psychological health of persons seeking HIV testing and documenting how psychiatric symptoms develop over time following receipt of an HIV positive diagnosis, has important implications for mental health care systems.

**Methods:**

We describe a study protocol to investigate: (1) the level of psychological distress and the prevalence of common mental disorders among persons seeking HIV testing; (2) the longitudinal development of psychiatric symptoms among persons diagnosed with HIV; and (3) the recommendations that can be made for mental health care systems to support persons seeking HIV testing and those newly diagnosed with HIV. In this longitudinal study quantitative and qualitative data are collected to document participants’ psychiatric symptoms, to determine whether they meet diagnostic criteria for a common mental disorder, and to explore the lived experiences of persons receiving an HIV positive test result. Data are collected at three time points; before HIV testing, and then again at 6 and 12 months post-testing.

**Discussion:**

Documenting the prevalence of common mental disorders among persons seeking HIV testing, and tracking the psychosocial support needs, psychological adjustment and psychosocial experiences of persons newly diagnosed with HIV, has important implications for the delivery of mental health care services and the design of integrated mental health care systems.

## Background

In many public health clinics, HIV testing has become an increasingly routine practice. As a result, large numbers of persons who have received an HIV positive test result and who qualify for treatment have been enrolled in antiretroviral therapy (ART) programs. In an effort to promote greater access to voluntary counseling and testing (VCT) for HIV, it has become practice in many countries, including South Africa, to establish non-medical testing sites and to de-couple HIV testing from other medical and mental health care services. Much of the literature in psychology has assumed that receiving an HIV positive test result may precipitate depressive disorders, symptoms of post-traumatic stress disorder, or other psychiatric symptoms, and that psychiatric or psychological treatment is indicated following a positive test [1–5]. However, empirical data about the baseline psychiatric condition of persons seeking HIV testing is absent. It is therefore unknown what the prevalence of common mental disorders^1^ is among persons seeking HIV testing and whether such individuals are already psychiatrically disordered or emotionally distressed prior to receipt of their HIV positive result. There are a number of plausible rival hypotheses to the dominant view that receipt of an HIV positive test result precipitates psychiatric illness, including: (1) that rates of psychopathology are high among persons seeking HIV testing; and (2) that prior mental health problems may be a superior predictor of post-HIV psychiatric disturbance than an HIV diagnosis in and of itself. The proposed study seeks to test these hypotheses. These issues have particular salience in low- and middle-income countries, like South Africa, where medical resources are scarce and data on the prevalence and course of mental disorders are needed to plan effective and integrated mental health care services.

### Depression and anxiety among persons with HIV

Little systematic epidemiological research has been conducted into the prevalence, incidence and duration of psychiatric disorders among South African patients living with a chronic illness in general, and those living with HIV in particular. Yet, as indicated in the international literature, disorders such as major depressive disorder (MDD) and generalized anxiety disorder (GAD) appear to be common among persons living with HIV [6–9]. In general, self-report measures have yielded higher prevalence rates than those determined by clinician-administered structured interviews [10]. However, both of these approaches show elevated rates of symptoms of depression and anxiety among persons living with HIV.

In Uganda, among a sample of 1017 HIV infected persons, 47% scored in the elevated range on the Center for epidemiological studies-depression scale, with those with low CD4 counts more than twice as likely to report elevated depressive symptoms than those with high CD4 counts [11]. On the other hand, in a comparison between HIV positive and negative men, lifetime prevalence of MDD were non-significantly different [12]. Among the same sample at 2 year follow-up, however, participants who were symptomatic with HIV disease were significantly more likely to have a depressive episode than asymptomatic HIV positive persons or HIV negative controls. Further, a lifetime history of depression increased the likelihood of depression post-HIV diagnosis. Atkinson et al. [12] concluded from their findings that symptomatic HIV disease, but not HIV infection itself, increases the risk of MDD and that a prior history of depression is a major risk factor for depression after diagnosis with HIV [12].

Among a sample of 996 Tanzanian women living with HIV, more than half reported symptoms of depression during the 2 year period in which they were followed [13]. Moreover, among this sample, depressive symptoms were associated with an elevated risk of disease progression and mortality, prompting Antelman et al. [13] to call for routine screening for depression and psychosocial interventions as part of normal HIV care.

Among a sample of 149 South African HIV-infected patients, 34.9% had MDD while 21.5% had dysthymic disorder as assessed by the MINI International Psychiatric Interview [14]. In a 6 month follow-up study, 26% of the sample continued to meet the criteria for a depressive illness [3]. Among another sample of South African AIDS patients, 33.4% reported symptoms of anxiety and depression, compared with 24.2% in a randomly selected community sample [15]. These studies recruited samples from peri-urban environments. In a study conducted with 465 patients enrolled in HIV care and treatment services in a major South African city, the prevalence of depression as assessed by the mini-international neuropsychiatric interview (MINI) was 14% [16]. Using the composite international diagnostic inventory (CIDI), Freeman et al. [17] found that among 900 HIV positive persons, 43.7% had a mental disorder and 11.1% had MDD. In these studies, samples were recruited after they had received an HIV diagnosis, and it was thus not possible to determine which participants might have had a pre-existing common mental disorder prior to receipt of the HIV positive test result.

### Post-traumatic stress disorder and HIV

The rate of posttraumatic stress disorder (PTSD) among individuals infected with HIV varies across studies as a result of variations in methods of assessment used to determine symptomatology, diagnostic criteria used to determine caseness, sample characteristics, and the diverse definitions of what qualifies as a traumatic event [18]. Despite both the varying estimates of PTSD prevalence, the available figures suggest that the rate of PTSD among persons living with HIV may be higher than in the general population [4]. For example, the estimated rate of DSM-III-R PTSD determined among an Australian sample of 61 HIV-positive homosexual and bisexual men was 30% [19]. Similarly, among a sample of 67 African American HIV-infected women and among an American sample of 41 HIV-positive women, the estimated rate of DSM-IV PTSD was 35 and 42%, respectively [20, 21]. Moreover, it was found that the estimated rate of DSM-IV PTSD among an American sample of 75 HIV-positive men and women was estimated at 64% [22]. Among a South African sample of recently diagnosed HIV-positive individuals, an estimated PTSD rate of 14.8 and 26% was determined at baseline and follow-up, respectively [3]. In general, the rate of PTSD reported in these studies is higher than that determined in the recently conducted South African Stress and Health Study [23]. In this nationally representative household survey conducted among 4351 South African adults, the lifetime prevalence of PTSD was estimated at 2.3% [23].

PTSD may either precede an HIV diagnosis due to previously experienced traumatic events, or may emerge post-HIV diagnosis as a result of the stress of being diagnosed with a life-threatening illness. The disorder may also result from the traumatic events experienced during the course of the illness. For example, in a study conducted among a sample of recently diagnosed HIV-positive individuals, 14.8% met criteria for PTSD, of which 36.4% reported that the index trauma was being informed of their HIV-positive diagnosis. Other index traumas constituted being raped (22.7%), being robbed or assaulted (13.6%), intimate partner violence (9.1%), having experienced a serious accident (9.1%) and the death of someone close to the individual (9.1%) [3, 14].

The level of PTSD has also been found to be positively correlated with the total number of traumatic life events experienced by HIV-positive individuals [20, 24]. For example, it was found that HIV-positive women who had experienced a greater number of traumatic life events had a higher percentage of probable full PTSD in comparison to those women who had experienced fewer traumatic life events [21].

It is our view that HIV does not constitute a stressor precipitating PTSD [16]. PTSD is a disorder of memory in which the symptoms of avoidance, intrusion, and physiologic hyperarousal are anchored to the traumatic event that occurred in the past. Concerns associated with receipt of an HIV positive test result are typically future-oriented, such as impending decline in health status, accessing treatment, finding information about the condition, and managing problems related to adherence. Certainly, distress may be highly common, but this is qualitatively different from trauma.

### Substance use disorders and HIV

Substance use has been shown to increase the risk of HIV infection. Hazardous use of alcohol, illicit drugs and intravenous drug use have been linked to the spread of the HIV epidemic, particularly in populations which are already vulnerable to infection [25, 26]. Evidence suggests that substance use adversely influences the effectiveness of ART and compromises treatment adherence among HIV infected persons [27, 28]. Substance use is also associated with increased risk of opportunistic infections, such as hepatitis [29]. Rates of substance use disorder (SUD) are higher among HIV infected groups when compared to the general population, with nearly 50% of persons living with HIV/AIDS reporting current or past histories of drug or alcohol disorders [30–32]. It has been suggested that treating substance use problems in persons with HIV/AIDS should be an important component of integrated HIV treatment, although doing so is not without considerable challenges [33].

### Common mental disorders among persons seeking HIV testing

While there has been considerable research on HIV and common mental disorders, little data has been accrued about the prevalence of common mental disorders among persons seeking HIV testing. There is also a dearth of empirical data showing that receipt of a diagnosis of HIV affects the mental health of patients. It is commonly assumed that receipt of a positive HIV test result is highly emotionally distressing and precipitates psychiatric illness. While this may indeed be the case for many people, data on psychological reactions of persons who receive an HIV positive test result is very sparse. The assumption that persons who receive a positive HIV test result necessarily experience intense psychological distress or psychiatric disturbance is untested in the present moment of the HIV pandemic. Some individuals may not react as negatively as is commonly assumed for at least four reasons. Firstly, in the present era of widespread public awareness programs regarding HIV risk, we believe that it is likely that persons who have engaged in high risk behavior are likely to have evaluated their risk level prior to testing. For this sub-group of the general public, a positive HIV test result may confirm what they have already suspected. Secondly, in the context of high HIV prevalence in many South African communities [34, 35] many persons undergoing testing may already know others in their social constellation, including sex partners, who have tested positive. For these persons, a positive HIV test is less likely to be as shocking, traumatic, or distressing as has been previously assumed [36]. Thirdly, widespread access to antiretroviral treatment has changed the texture of the experience of living with HIV from impending physical decline and death to that of a chronic illness. Fourthly, difficult life circumstances characterized by poverty, unemployment, and food insecurity make HIV one of a range of life stressors, but not necessarily the most salient one in low resource settings. While the majority of persons receiving an HIV positive test result may indeed be distressed, it is likely that HIV-related distress may ameliorate over time. Thus it is necessary to document the psychological reactions to an HIV diagnosis and to track, over an extended period of time, the psychosocial experiences of persons receiving an HIV positive result.

In addition to the lack of epidemiological data about the prevalence of common mental disorders among persons seeking HIV testing, there have been no attempts to validate commonly used psychometric instruments among populations of people seeking HIV testing. Similarly, no clinically significant cut-off points have been established for commonly used psychiatric symptom checklists, among persons seeking HIV testing. This gap in the literature hinders efforts to screen for common mental disorders among persons seeking HIV testing, using instruments such as the Beck depression inventory (BDI), Beck anxiety inventory (BAI), Hopkins symptom checklist (HSC), posttraumatic stress disorder symptom scale (PTSD-SS), alcohol use disorders identification test (AUDIT), and drug use disorder identification test (DUDIT).

It is within this context that we describe a protocol for a study in the Western Cape province of South Africa which has the following six objectives:

1. To determine the level of psychological distress (as measured by the HSC), and the prevalence of MDD, GAD, PTSD, and substance use disorders [as measured by the Structured Clinical Interview for DSM-5 (SCID-5)] among persons who are seeking HIV testing.
2. To determine the sensitivity, specificity, and positive and negative predictive values of the BDI, BAI, PTSD-SS, AUDIT, and DUDIT in predicting MDD, GAD, alcohol use disorder and drug use disorder, respectively among persons seeking HIV testing.
3. To document fluctuations in the level of psychological distress and the development of psychiatric symptoms in the 12 month period following receipt of an HIV positive diagnosis.
4. To determine if there are significant differences between HIV positive and HIV negative individuals, on self-report measures of common mental health problems, over the course of 1 year following HIV testing.
5. To document the lived experience of a group of individuals from low income settings in the 12 month period following receipt of an HIV positive diagnosis, in order to learn from them about: the specific resource challenges they experience (such as food insecurity, housing, employment, and social and family support); their psychosocial concerns; their perception of the barriers to receiving appropriate care; and their access to health care services and psychosocial support.

### The South African context

Data collected in the South African *2012 HIV Prevalence, Incidence and Behaviour Survey,* using a nationally representative sample of 38,431 respondents from 11,079 households, reported an HIV prevalence rate of 12.2% (95% CI 11.4–13.1), a statistically significant increase from 10.6% in 2008 (p < 0.001) [37]. In this household survey HIV prevalence rates were found to be higher among females (14.4%) than males (9.9%), and adults aged 25–49 years were most affected (25.2%, 95% CI 23.2–27.3). ART exposure doubled from 16.6% in 2008 to 31.2% in 2012 (p < 0.001), partly as a result of better access to VCT. HIV testing is considered to be an important entry point to a comprehensive package of care for HIV and AIDS prevention and treatment. In response to the HIV epidemic, the South African government and numerous international aid organizations have invested resources in VCT; by 2009 the state had established more than 450 VCT centers with more than 800 counselors around the country [38]. As a result, population-level VCT in South Africa is among the highest globally, with a large proportion of South Africans knowing their HIV status [37].

For a range of historic, political and socio-economic reasons, South Africa has a poor health system, which is characterized by a lack of integration, poor access, and serious resource constraints [39, 40]. Primary health care remains predominantly biomedical in its orientation with a lack of person-centered, integrated health care [41, 42]. Mental health care resources are particularly scarce in South Africa [43], and there is a significant mental health treatment gap; an estimated 15.9% of adults with mental health problems report receiving treatment [44]. A population based study, indicated that the 12-month prevalence of DSM-IV disorder was 16.5%, with the most common disorders being agoraphobia (4.8%), MDD (4.9%), and alcohol abuse or dependence (4.5%) [44].

## Methods

This exploratory mixed method study employs quantitative research methods (to elicit measures of psychiatric symptoms) and qualitative methods (to produce a description of the lived experiences of individuals who receive an HIV positive test result). The research follows a longitudinal design. A total of 500 participants will be tracked over the course of 1 year. We calculated the minimum sample size needed to provide sufficient power for the proposed regression analysis, given the number of anticipated predictor variables we intend to include in our analysis.

### Participants

Participants in the study will be persons who have presented for HIV testing at non-medical HIV testing sites in peri-urban communities around Cape Town. Only individuals who are conversant in English or Afrikaans will be recruited. The only exclusion criteria are the presence of active symptoms of a psychotic illness; being under the age of 18; and being unable to give informed consent to participate in the study.

### Procedure

Persons seeking HIV testing will be invited to enroll in the study prior to their HIV test. Once they register at the clinic reception, they will be handed a flyer informing them of the study and inviting them to meet with a researcher in a private room. Persons who agree to meet with the researcher will be informed about the study in person and will be invited to participate in the study before undergoing HIV testing. It will be explained to participants that the study will involve three data points over the course of 1 year and that they will be contacted 6 months and 1 year later.

Participants will be asked to provide their contact details, including mobile telephone numbers, email addresses (where possible), and home addresses. Those who agree to participate will be asked to complete an informed consent form. Participants will receive a grocery voucher worth R50 as a token of gratitude for completing the questionnaire battery at each time point as well as payment for transport of R20 at the 6 and 12 month time points. They will then be asked to participate in a psychiatric interview and complete a battery of questionnaires (time 1).

### Testing procedures

Participants will proceed with HIV testing and after receiving their results will receive routine post-test counseling provided by the clinic. The HIV status (as determined by the HIV test) for each participant will then be recorded.

After 6 months (time 2) all participants will be telephoned and asked to attend a data collection session at the testing site. The data collection session will be scheduled at specific times so that large groups of participants may attend and complete their questionnaires simultaneously. Participants will be given a choice of times that are most convenient for them. This procedure will be repeated again 12 months after HIV testing (time 3).

### Data collection

Data will be collected at three points during the study, as described below.

#### Time 1: At the time of HIV testing

1. Data will be collected for the following variables:
2. Demographic variables including employment status, housing, food insecurity, and family living circumstances.
3. Common mental disorders: MDD, GAD, PTSD, and SUD will be assessed using the research version of the SCID-5. We will use a SCID-5 that has been adapted to comply with the changes to diagnostic criteria that came into effect with the introduction of the DSM-5.
4. Levels of psychological distress will be assessed (using the HSC), and symptoms of MDD, GAD, PTSD, and SUD will be assessed (using the BDI, BAI, PTSD-SS, AUDIT, and DUDIT). These psychometric instruments were selected because they have good psychometric properties [45–48], and because they are widely used in the field of HIV research, which will enable easy comparison of findings in this study with the existing literature. During the administration of these instruments, participants will be reminded that they are being asked to reflect on how they felt in the past weeks, and not simply how they are feeling right now. Participants with low levels of literacy will be assisted in completing the paper-based self-report questionnaires by a trained data collector, who will read the questions to the participant and record their responses.

#### Time 2: Six months after HIV testing

Data will be collected for all 500 participants using a combination of psychometric instruments and self-report questionnaires. Participants with low levels of literacy will be assisted by trained data collectors in completing the paper based self-report measures. The following data will be collected:

1. Symptoms of depression, anxiety, posttraumatic stress, and alcohol and drug use, as measured by the BDI, BAI, HSC, PTSD-SS, AUDIT, and DUDIT.
2. Socio-economic circumstances: food security, housing circumstances, employment status, and access to medical treatment.

Qualitative data will also be collected via a semi-structured interview for a sub-sample of 20 individuals (or until saturation) randomly selected from each of the following sub-groups: (1) persons living with HIV who also meet the diagnostic criteria for a mental disorder as identified by data collected at time 2; and (2) persons living with HIV who do not meet the diagnostic criteria for a mental disorder as identified by data collected at time 2.

During these interviews participants will be asked to describe their living circumstances, subjective experience of quality of life, general health status, subjective experience of psychological wellbeing, adjustment to HIV diagnosis, disclosure of HIV status in the past 6 months, support systems, experience of stigma, coping strategies, access to medical health care (including HIV treatment) and psychosocial support services. Participants will also be asked where they have accessed medical and psychosocial care in the preceding 6 months, including contact with traditional healers and other complementary and alternative health care providers. Participants will be asked to describe their current medical and psychosocial support needs and their perception of barriers to receiving care.

#### Time 3: Twelve months after HIV testing

Data will be collected for the following variables for all 500 participants using a combination of psychometric instruments and self-report questionnaires:

1. Symptoms of depression, anxiety, posttraumatic stress, and drug and alcohol use as measured by the BDI, BAI, HSC, PTSD-SS, AUDIT and DUDIT.
2. Socio-economic circumstances: Food security, housing circumstances, employment status, and access to medical treatment.

Once again participants with low levels of literacy will be assisted in completing the questionnaires.

Qualitative interviews will be conducted with two randomly selected sub groups of 20 participants. The same semi-structured interview schedule employed at time 2 will be utilized to collect qualitative data from: (1) persons living with HIV who also meet the diagnostic criteria for a mental disorder (as assessed at time 3); and (2) persons living with HIV who do not meet the diagnostic criteria for a mental disorder (as assessed at time 3).

### Data analysis

Table 1, below, indicates how data will be analyzed to achieve each of the study objectives:

**Table 1:** Data analysis method

Study objective	Data analysis method
*Objective 1*: To determine the level of psychological distress (as measured by the HSC), and determine the prevalence of MDD, GAD, PTSD, and SUD (as measured by the SCID-5) among persons who are seeking an HIV test	Descriptive statistics will be used to determine the level of psychological distress, and prevalence estimates for common mental disorders, using a confidence interval of 95%. Rates of comorbidity will also be calculated to determine the number of individuals who meet diagnostic criteria for two or more disorders
*Objective 2:* To determine the sensitivity, specificity, and positive and negative predictive values of the BDI, BAI, PTSD-SS, AUDIT and DUDIT in predicting MDD, GAD, PTSD, and SUD, respectively among persons seeking HIV testing	The sensitivity, specificity and positive and negative predictive values of self-report measures, will be calculated using receiver operator curve (ROC) analysis [49]. By determining the area under the curve for each of the self-report measures, we will be able to establish the optimal cut-score on the various self-report measures, using the SCID-5 as the gold standard
*Objective 3:* To document fluctuations in the level of psychological distress and the development of psychiatric symptoms in the 12 month period following receipt of an HIV positive diagnosis	Repeated measures of ANOVA for scores obtained on the HSC, BDI, BAI, PTSD-SS, AUDIT, and DUDIT, at times 1, 2 and 3, will be used to establish if there are significant fluctuations in levels of psychological distress and symptoms of psychiatric disorders over the 12 month period following receipt of an HIV positive diagnosis, using a confidence interval of 95%. Mixed-effects regression models will be used where appropriate to take account of both fixed effects and random effects, on the repeated measures of psychiatric symptoms over the three time points [50]
*Objective 4:* To determine if there are significant differences between HIV positive and HIV negative individuals, on self-report measures of common mental health problems, over the course of 1 year following HIV testing	A t-test will be used to determine whether a significant difference exists between HIV positive and negative persons on the means of their scores on the BDI, BAI, PTSD-SS, AUDIT and DUDIT. These comparisons will be made at 6 and 12 months after receiving an HIV test
*Objective 5:* To document the lived experience of a group of individuals from low income settings in the 12 month period following receipt of an HIV positive diagnosis, in order to learn from them about: the specific resource challenges they experience (such as food insecurity, housing, employment, and social and family support); their psychosocial concerns; their perception of the barriers to receiving appropriate care; and their access to health care services and psychosocial support	Interpretive phenomenological analysis (IPA) will be used to elicit a thick description of the participants’ lived experiences of adjusting to an HIV positive status. Themes will be identified and organized into over-arching themes which provide insight into how individuals understand, adjust to and manage their HIV positive status. Similarly, the interviews will be used to provide insight into specific resource challenges they experience, their psychosocial concerns, medical and psychosocial support needs, access to care and perceived barriers to receiving care. The themes elicited in the interviews with participants who do not meet diagnostic criteria for a common mental disorder, will be compared with the themes emerging from the interviews with patients who do meet criteria for a common mental disorder. This comparison between the lived experience of the two groups will hopefully provide insight into why some individuals develop psychiatric symptoms following receipt of an HIV positive test result, while others do not. We have chosen to use IPA to analyze the qualitative data as this is a well-established and structured approach which is well suited to exploring people’s lived worlds; this method has also been extensively used in health psychology research to document patients’ experience of illness and recovery [51]

### Ethical considerations

Permission to conduct this study has been obtained from the Stellenbosch University Health Research Ethics Committee and institutional permission was obtained from the Western Cape Department of Health and non-medical testing sites where data will be collected. Written informed consent will be obtained from all participants prior to data collection. All data collected will be securely stored in a manner that protects participants’ privacy and right to confidentiality.

Participants who receive an HIV positive test result are likely to become distressed. However, their distress is unrelated to study participation and they will receive post-test counseling as part of routine care at the testing site. Participants identified as having clinical significant psychiatric symptoms will be referred for mental health services at the clinic or in the community. These referrals to mental health services will be handled sensitively to minimize the risk of exacerbating psychological distress or stigmatizing participants. Typically, access to mental health care services in this community is limited, but we have secured services for participants who require them in community based NGOs and at a local university psychology clinic which provides services to individuals who cannot access care in other settings.

There are no major risks associated with this study as participants will only be asked to complete a paper and pencil questionnaire battery, and answer questions about their lived experience and symptoms of psychopathology. There is a small possibility that some participants might become distressed by completing the questionnaire battery. These participants will be referred for psychological counseling either at the clinic or at a community counseling center.

There are no direct benefits to participants for taking part in this study as it is a descriptive and not an intervention study. However, persons who meet the diagnostic criteria for a common mental disorder or who score in the clinical range on the self-report measures will be referred for mental health services at an appropriate community clinic. Further, answering questions related to psychological symptoms may alert participants to their psychological state and thus increase self-knowledge.

## Discussion

The data collected from this project promises to provide important information about: (1) the level of psychological distress and the prevalence of common mental disorders among persons seeking HIV testing; (2) the sensitivity, specificity, and positive and negative predictive values of commonly used psychometric instruments in diagnosing common mental disorders among persons seeking HIV testing; (3) fluctuations in the level of psychological distress and psychiatric symptoms in the 12 month period following an HIV positive diagnosis; (4) the extent to which there are significant differences between HIV positive and HIV negative individuals, on self-report measures of common mental health problems, over the course of 1 year following HIV testing; and (5) the lived experience of a group of individuals from low income settings in the 12 month period following receipt of an HIV positive diagnosis. The findings have the potential to refute or confirm the commonly accepted (and as yet untested) idea that receipt of an HIV diagnosis is in and of itself a psychological trauma that precipitates psychological distress and mental illness. Crucially, the findings will also allow us to make empirically supported recommendations concerning an integrated package of care, including mental health care services, for persons seeking HIV testing and those newly diagnosed with HIV that may enhance engagement with treatment and influence adherence. Furthermore, establishing the sensitivity, specificity, and positive and negative predictive values of psychometric instruments (such as the BDI, BAI, PTSD-SS, AUDIT and DUDIT) for use among persons seeking HIV testing, would be useful for future research and clinical practice which entails screening or assessing the psychiatric status of persons seeking HIV testing, monitoring the trajectory of psychiatric symptoms following receipt of an HIV test, and planning appropriate context-sensitive mental health care services.

In spite of the potential value of this proposed study, there are a number of possible limitations. Firstly, the study design relies on a convenient self-selected sample of people. Although it is acceptable to make use of non-probability sampling in studies which seek to collect information from hard to reach populations, the use of such an approach will limit the generalizability of the findings [52]. Secondly the diagnostic criteria in the SCID-5 are very strict compared to the CIDI. Consequently, we may not diagnose some participants with a common mental disorder, even though they would have met criteria based on the CIDI. Prevalence estimates obtained in this study may thus be under-estimates. Thirdly, the SCID is criterion based rather than guideline based; there is thus very limited leeway to interpret symptoms or exercise clinical judgment in the assessment of participants. This too may lead to under-estimates of prevalence rates. Fourthly, there is a danger that in both the structured clinical interviews and the qualitative interviews, participants might be influenced by the demand characteristics of the interview situation, thus endorsing symptoms and/or editing what they report, in order to appear compliant or meet the expectations of the interviewer. The risk of this is compounded by the fact that participants receive compensation for taking part in the interviews. Finally, the study design requires assessments at three time points, using a wide variety of instruments. This will be time consuming, costly and could be onerous for participants, which raises questions about the feasibility of the study. It is, however, necessary to include this full battery of instruments in order to meet the objectives of the study and provide a comprehensive assessment of the mental health care needs of persons seeking HIV testing and those newly diagnosed with HIV. We intend trying to overcome this potential limitation by providing incentives to participants for completing questionnaires, and arranging the follow up testing to be done in groups at times convenient to participants.

We anticipate that there might well be difficulties with follow-up and attrition, given the longitudinal research design of the study and the intention to collect data 6 and 12 months post HIV testing. It is difficult to know what the rate of attrition will be and how difficult it may be to trace participants for follow-up. Attrition in longitudinal studies is a well-documented problem in epidemiological research [53, 54], particularly in low- and middle-income countries [55]. Even though we intend to collect contact numbers and residential addresses for participants, it is well known that people living in low resource communities in South Africa are very mobile; they move homes frequently and change their cell phone numbers periodically.

## Conclusion

Contact with the health care system while seeking HIV testing provides an opportunity for psychological intervention and the early detection and treatment of psychopathology. Hitherto this opportunity for intervention has been missed, in part because HIV testing has been uncoupled from the provision of other medical and psychosocial support services in countries like South Africa. In an effort to provide greater access to HIV testing in South Africa, a large number of non-medical HIV testing sites have been established, thus contributing to and perpetuating an unintegrated health care system. This lack of integration of mental health care services within HIV testing sites, is premised on the untested assumption that psychological distress is a consequence of receiving an HIV positive diagnosis. Conducting studies like the one described in this protocol could help to establish if there is a need for better integration of mental health services, especially screening for mental disorders and referral of those persons who are in need of services, as part of efforts to strengthen mental health care systems in low- and middle-income countries. Understanding the trajectory of psychological adjustment following receipt of an HIV diagnosis will also help plan better mental health care services to support newly diagnosed individuals.

## Abbreviations

AIDS: acquired immune deficiency syndrome
ART: antiretroviral therapy
AUDIT: alcohol use disorders identification test
BAI: Beck anxiety inventory
BDI: Beck depression inventory
DSM: diagnostic and Statistical Manual
DUDIT: drug use disorder identification test
GAD: generalized anxiety disorder
HIV: human immunodeficiency virus
HSC: Hopkins symptom checklist
IPA: interpretive phenomenological analysis
MDD: major depressive disorder
MINI: mini-international neuropsychiatric interview
PTSD: posttraumatic stress disorder
PTSD-SS: posttraumatic stress disorder symptom scale
ROC: receiver operator curve
SCID-5: structured clinical interview for the DSM-5
SUD: substance use disorder
VCT: voluntary counseling and testing

## Footnotes

1 Common mental disorders are defined, for the purposes of this study, as psychiatric conditions which are most frequently associated with HIV infection. These include depressive disorders, anxiety disorders, trauma-related disorders, and substance use disorders.
